# Factors influencing government insurance scheme beneficiary acceptance of the gatekeeper policy: a cross-sectional study in Wuhan, China

**DOI:** 10.1186/s12913-018-3010-4

**Published:** 2018-04-04

**Authors:** Wenzhen Li, Dongming Wang, Yong Gan, Yanfeng Zhou, Yawen Chen, Jing Li, Naomiem Kkandawire, Sai Hu, Yan Qiao, Zuxun Lu

**Affiliations:** 1 Department of Social Medicine and Health Management, School of Public Health, Tongji Medical College, Huazhong University of Science and Technology, No. 13 Hangkong Road, Wuhan, 430030 Hubei People’s Republic of China; 2 Department of Occupational & Environmental Health, School of Public Health, Tongji Medical College, Huazhong University of Science and Technology, Wuhan, 430030 China; 3 Wuhan Hospital for the Prevention and Treatment of Occupational Diseases, Wuhan, 430030 China

**Keywords:** Acceptance, Patient satisfaction, Gatekeeper policy

## Abstract

**Background:**

Gatekeeper policy, requiring a patient to visit a primary care provider first, and the patient needs to get his or her primary care provider’s referral before seeing a specialist or going to a hospital, has been implemented in China for about ten years, and it is necessary to assess the patients’ acceptance of gatekeeper policy and to explore the factors influencing patients’ acceptance.

**Methods:**

A cross-sectional study with 1162 respondents was conducted between July and September 2015 at four community health centers (CHCs) in Wuhan, China. Face-to-face interview was used to collect information on demographics, acceptance of the gate keeper policy and satisfaction with community health services. Patients’ satisfaction with community health service was evaluated using the European Patients Evaluate General/Family Practice scale and binary logistic regression model was used to examine the factors influencing patients’ acceptance of community health services as gate keepers.

**Results:**

A total of 512 (43.06%) patients accepted gatekeeper policy. Mandatory reimbursement provision (OR: 1.63, 95% CI: 1.23–2.15), patient satisfaction with the aspects of medical care (OR: 1.92, 95% CI: 1.12–3.29) and organization of care (OR: 1.66, 95% CI: 1.05–2.62) were associated with acceptance of gatekeeper policy, after adjusting for potential confounders. Moreover, young people (OR: 0.35, 95%CI: 0.22–0.56) seemed to be more reluctant to accept the policy, when compared with the elder.

**Conclusions:**

Our study suggests that mandatory reimbursement provision greatly affects patients’ acceptance of gatekeeper policy, therefore, the policy-maker should pay attention to the negative effect of its mandatory reimbursement provision on patients’ acceptance of the policy. However, improving the aspects of medical care and organization of care will contribute to implementation of gatekeeper policy.

**Electronic supplementary material:**

The online version of this article (10.1186/s12913-018-3010-4) contains supplementary material, which is available to authorized users.

## Background

China achieved near-universal health insurance coverage before economic reform, and about 90% of residents were covered by Cooperative Medical System in rural areas, while in urban areas, almost everyone was covered by Government Insurance Scheme (GIS) and Labor Insurance Scheme [1]. The GIS which covered government employees, teachers, and retirees was funded by government budgets. People covered by GIS spent only a few amount of money or were free of charge on their medical care. However, they were required to abide to gatekeeper policy. In China, the gatekeeper policy is around compulsory reimbursement provision: if a patient is subjected to the gatekeeper policy, he or she could get all or most of compensation and if the patient seeks care from a specialist or a hospital without referral, he or she has to pay all the charges by him or herself.

By 1980s, China had converted to the free-market economy and the health system was in the front. One of the most changes was the establishment of social medical insurance (SMI) scheme that combined individual medical savings accounts and hospitalization insurance, and GIS was replaced by SMI in most areas [2]. People covered by SMI could freely choose any medical institution for medical care without restrictions. In the meantime, the community healthcare network was disintegrated and the China’s health-care delivery became hospital-centered and fragmented [3]. Unfortunately, the market-reform experiments made the residents had no faith in health service facilities, especially in primary health institutions. Discontent with insufficient access to health service and expensive medical cost [4] threatened social stability and deteriorated the doctor-patient relationship [5].

Chinese government had recognized the importance of community health service (CHS) and made great efforts to improve it [3]. However, patients still preferred to seek medical care from the best health care facilities, which worsened the irrational and wasteful health care delivery system. In order to channel patients to primary health service institutions, the Chinese government has taken various measures, such as providing more convenient outpatient services or lowering the cost of drug at community health centers (CHCs), to appeal to patients, but the effect was apparently unsatisfactory. Gatekeeper policy has been widely implemented in tax-funded health systems, such as those in the United Kingdom and Spain, and in other social health insurance systems, such as those in Switzerland and the Netherlands [6–9].This policy orients a health system towards primary care by channeling patients and health resources to primary care providers [10, 11]. Lessons learned from developed countries, Chinese government issued a directive endorsing gatekeeper policy. Essentially, the gatekeeper system requires a patient to visit a primary care provider first, and the patient needs to get his or her primary care provider’s referral before seeing a specialist or going to a hospital. This policy has proved to be an effective policy [12, 13], aiming to establish a well-arranged health service system.

So far, many pilot programs have been launched among special population such as the elderly, migrant workers covered by different health insurance schemes. However, it is difficult to evaluate properly patients’ acceptance of gatekeeper policy among the pilot population because they could not experience the policy deeply in a short time. Previous study showed that patients’ willingness for visiting CHCs is high [14] and another study indicated that patients’ satisfaction with the gatekeeper policy was low [15], however, these study were conducted among migrant workers and they experience the policy only for 15 years. In fact, people covered by GIS have experienced gatekeeper policy for a long time, and their attitude towards the policy would be suitable and available for the assessment of the gatekeeper policy in China. Currently, the GIS which covers government employees and university teachers still remains in only a few cities, such as in Wuhan city. This study is the first attempt to assess the acceptance of gatekeeper policy as well as to explore its impact factors.

## Methods

This cross-sectional study was conducted at CHCs in Wuhan, Hubei province, from July to September in 2015. Four CHCs that gathered people with GIS were randomly selected and 250~ 300 outpatients with GIS at each CHC were randomly interviewed. Patients younger than 18 years old were excluded. A total of 1200 questionnaires were distributed and 1162 were included in the study, of which 38 questionnaires were excluded for missing data. The overall response rate was 96.83%.

The questionnaire contained questions about the patient’s gender, age, marital status, educational level, income, health status, chronic diseases, whether attendance to CHCs owing to mandatory reimbursement, and acceptance of the gatekeeper policy. Patients’ health scores were determined by self-reported, and 0 score represented the worst health status while 100 score represented the best health status. In addition, the European Patients Evaluate General/Family Practice (EUROPEP) instrument was used to assess patient satisfaction with CHS. The EUROPEP scale included five dimensions, that is, doctor-patient relationship, medical care, information and support, organization of care (continuity and cooperation) and accessibility. The satisfaction were marked using a 5-point Likert scale ranging from “poor” to “excellent”, with “acceptable” as the middle value.

Information was collected by trained interviewers through face to face interview. The patient who was about to leave the CHC was provided with an overview of our research, and then completed the survey questionnaire if he or she was willing to participate. Each questionnaire was checked carefully by investigators in field. The data was double-blindly entered into the database by two trained investigators using EpiData 3.1 software.

Binary logistic regression analyses were used to assess the effect of mandatory reimbursement provision and patients’ satisfaction on acceptance of gatekeeper policy, and patients’ acceptance of gatekeeper policy was assessed using a single item “would you like to accept the gatekeeper policy?” The stepwise selection method was used to include/exclude variables in/from the logistic regression model (level for selection and elimination: *P* = 0.05 and *P* = 0.10, respectively). The Chi-squared (χ^2^) test was used to compare the socio-demographics characteristics and patients’ satisfaction in two groups (acceptable vs non-acceptable). For each item of EUROPEP scale, patient’s evaluation was regarded as positive if it was one of the two most approbatory categories (“4” or “5”). An evaluation of a dimension was grouped into 100%, 50~ 99% or 0~ 49% according to the proportion of positively assessed items in that dimension. In the present analysis, the most positive assessments showed patients who marked 100% of the answered questions in one of the two most positive answering categories, the neutral assessments showed patients who marked 50%~ 100% of the answered questions in one of the two most positive answering categories, and the poor assessments showed patients who marked less than 50% (0–49%) of the answered questions in one of the two most positive answering categories [16, 17]. Age, gender, marital status, educational background, income of family, health score and chronic condition were considered as confounding factors in the regression. The reliability of EUROPEP was assessed with Cronbach’s α ($$ \upalpha =\frac{k}{k-1}\Big(1-\sum \limits_{i=1}^k\raisebox{1ex}{${S}_i^2$}\!\left/ \!\raisebox{-1ex}{${S}_p^2$}\right. $$) and the validity of EUROPEP was evaluated using factory analysis and was assessed by Kaiser-Meyer-Olkin (KMO). The reliability and validity were high (Cronbach’s α = 0.96 and KMO = 0.96). The data were analyzed using SPSS, version 18.0 (SPSS Inc., Chicago, Ill). All differences were tested using two-tailed tests and a *P-*value of 0.05 was considered statistically significant.

## Results

A total of 1162 participants (512 accepted the gatekeeper policy [AGP] and 650 did not accept the policy [NAGP]) were included in this study. Distribution of socio-demographic characteristics and attendances to CHCs because of mandatory reimbursement provision were presented in Table 1. Significant differences were found in the distribution of age and chronic conditions between AGP and NAGP. 64.4% AGP were over 60 years old while the percentage of NAGP patients was only 45.5%. 63.9% patients who attended CHC for treatment due to mandatory reimbursement provision were not willing to accept gatekeeper policy and 43.1% patients who voluntarily attended CHC for treatment could accept gatekeeper policy.

**Table 1:** The characteristics of the study population according to acceptability

	AGP		NAGP		χ^2^	*P*-value
	n(512)	%	n(650)	%		
Age						
18~ 40	47	9.18	148	22.77	53.31	< 0.01
40~ 60	135	26.37	206	31.69		
≥ 60	330	64.45	296	45.54		
Gender						
Male	272	53.13	316	48.62	2.33	0.13
Female	240	46.87	334	51.38		
Marital status						
Married	453	88.48	577	88.77	0.02	0.88
Single	59	11.52	73	11.23		
Educational Background						
Primary school and below	13	2.54	12	1.85	4.07	0.13
Middle school	94	18.36	94	14.46		
College degree and above	405	79.10	544	83.69		
Income of family monthly						
< 3000	24	4.69	21	3.23	4.22	0.12
3000~ 5000	155	30.27	172	26.46		
≥ 5000	333	65.04	457	70.31		
Health score						
≥ 80	251	49.02	339	52.15	2.50	0.29
60~ 80	202	39.45	253	38.92		
< 60	59	11.52	58	8.92		
Chronic conditions						
No	129	25.20	251	38.62	23.44	< 0.01
Yes	383	74.80	399	61.38		
Visit to CHC owing to mandatory reimbursement provision						
no	221	43.16	235	36.15	5.90	0.02
yes	291	56.84	415	63.85		

Table 2 showed that the differences in five dimensions of EUROPEP between AGP and NAGP were statistically significant. The dimensions of the EUROPEP included doctor-patient-relationship (6 items), medical care (5 items), information and support (4 items), organization of care (2 items) and accessibility (6 items). 38.09%, 34.96%, 27.73%, 46.29% and 7.62% AGP most positively assessed the five dimensions, respectively; while more than 50% NAGP poorly assessed all the dimensions.

**Table 2:** Distribution of patients’ assessment of CHS according to acceptability

	AGP		NAGP		χ^2^	*P*-value
	n	%	n	%		
Doctor-patient-relationship(6 items)						
Most positive assessments^a^	195	38.09	153	23.54	62.61	< 0.01
Neutral assessments^b^	154	30.07	140	21.54		
Poor assessments^c^	163	31.84	357	54.92		
Medical care(5 items)						
Most positive assessments	179	34.96	115	17.69	78.32	< 0.01
Neutral assessments	100	19.53	73	11.23		
Poor assessments	233	45.51	462	71.08		
Information and support(4 items)						
Most positive assessments	142	27.73	108	16.62	83.64	< 0.01
Neutral assessments	160	31.25	102	15.69		
Poor assessments	210	41.02	440	67.69		
Organization of care(2 items)						
Most positive assessments	237	46.29	174	26.77	94.31	< 0.01
Neutral assessments	151	29.49	138	21.23		
Poor assessments	124	24.22	338	52.00		
Accessibility(6 items)						
Most positive assessments	39	7.62	33	5.07	45.24	< 0.01
Neutral assessments	171	33.40	113	17.38		
Poor assessments	302	58.98	504	77.54		

^a^Patients who marked 100% of the answered questions in one of the two most positive answering categories

^b^Patients who marked 50%–99% of the answered questions in one of the two most positive answering categories

^c^Patients who marked less than 50% (0–49%) of the answered questions in one of the two most positive answering categories

We explored the factors influencing patients’ acceptance of gatekeeper policy and the results were showed in Table 3. Compared with patients who go to CHCs for treatment owing to mandatory reimbursement, patients who go to CHCs voluntarily was associated with acceptance of gatekeeper policy (OR: 1.63, 95% CI 1.23–2.15), and the dimension of medical care was also associated with acceptance of gatekeeper policy (OR: 1.92, 95%CI: 1.12–3.29), when controlling for confounding factors. In addition, the aspect of organization of care also affected patients’ acceptance of gatekeeper policy (OR: 1.66, 95% CI: 1.05–2.62 for most positive assessments and OR: 2.10, 95% CI: 1.45–3.04 for neutral assessments vs poor assessments), controlling for other confounding factors. Besides, age was also associated with acceptance of gatekeeper policy (OR: 0.35, 95%CI: 0.22–0.56 for 18~ 40 and OR: 0.61, 95% CI: 0.44–0.86 for 40~ 60 vs over 60).

**Table 3:** Factors affecting acceptation of gatekeeper policy among participants using binary logistic regression

Variables	OR	95%CI	*P*-value
Visit to CHC because of mandatory reimbursement provision (ref. = yes)			
no	1.63	1.23–2.15	< 0.01
Doctor-patient-relationship^a^			
Most positive assessments	0.93	0.57–1.51	0.77
Neutral assessments	1.15	0.78–1.70	0.48
Medical care^a^			
Most positive assessments	1.92	1.12–3.29	0.02
Neutral assessments	1.39	0.89–2.18	0.15
Information and support^a^			
Most positive assessments	1.20	0.68–2.12	0.53
Neutral assessments	1.53	1.0–2.34	0.05
Organization of care^a^			
Most positive assessments	1.66	1.05–2.62	0.03
Neutral assessments	2.10	1.45–3.04	< 0.01
Accessibility^a^			
Most positive assessments	1.19	0.63–2.26	0.60
Neutral assessments	1.26	0.86–1.82	0.23
Age(ref. = more than 60)			
18~ 40	0.35	0.22–0.56	< 0.01
40~ 60	0.61	0.44–0.86	< 0.01
Gender(ref. = male)			
Female	0.87	0.66–1.14	0.31
Marital status(ref. = married)			
Single	0.89	0.57–1.38	0.60
Educational Background (ref. = College degree and above)			
Primary school and below	0.95	0.37–2.41	0.91
Middle school	0.94	0.64–1.40	0.77
Income of family monthly (ref. = more than 5000)			
< 3000	0.94	0.47–1.96	0.91
3000~ 5000	0.97	0.70–1.34	0.86
Health score(ref. = less than 60)			
≥ 80	1.26	0.77–2.04	0.36
60~ 80	1.14	0.71–1.83	0.58
Chronic conditions(ref. = yes)			
No	0.77	0.55–1.07	0.12

^a^the reference group was poor assessments group

## Discussion

Gatekeeper policy along with health insurance programs is a critical policy in orienting a health system towards primary health care [18, 19]. There have been a substantial amount of researches about the effect of gatekeeping [12] on health- and patient-related outcomes [20], satisfaction with care [21], quality of care [22] and utilization of health care [23], but few studies about patients’ acceptance of gatekeeping were conducted. Our study is an important supplement in the research field of gatekeeper policy.

The research was conducted among the population covered by GIS, among whom the policy had been implemented for many years, and the differences between the attendances of patients to CHC for treatment owing to mandatory reimbursement provision in the two groups suggested that the compulsory policy about reimbursement might be associated with patients’ acceptance. In addition, we used the EUROPEP scale [17], an internationally-accepted questionnaire with high reliability and validity (Cronbach’s α = 0.96 and KMO = 0.96), to assess patient satisfaction with CHS, and the distribution differences of satisfactions with CHS suggested that patients’ satisfaction with CHS might be also associated with their acceptance (Additional file 1).

Furthermore, we conducted the regression analysis to examine the above speculations, and the results suggested that attendance to CHC for treatment owing to mandatory reimbursement provision was independently associated with patients’ acceptance of gatekeeper policy, when controlling for patients’ satisfaction and socio-demographic characteristics. It implied that the policy-maker should pay more attention to the effect of mandatory provision on patients’ acceptance of gatekeeper policy. Indeed, the mandatory reimbursement provision restricted patients’ choices in a certain extent, and thus they may be not willing to accept the gatekeeper policy. However, the policy-makers may have to compare the advantage and disadvantage of mandatory provision, simplifying the procedure of reimbursement or making the referral channels unimpeded may reduce the impact effectively, but they need further verification.

Another important finding was that not all dimensions of patients’ satisfaction with CHS affected their acceptance of the policy. Generally, when a patient was asked “why he or she was not subject to gatekeeper policy”, he/she may answer “I’m not satisfied with CHS”, however, it was complex and multidimensional in the diagnostic and therapeutic procedures for patients’ treatment in CHCs. Therefore, to determine the aspect that played a more prominent role in affecting patients’ acceptance was of great importance. Our study suggested that only the aspects of medical care and organization of care were positively associated with acceptance of policy. Therefore, managers and general practitioners should pay more attention to the two aspects of care when implementing the gatekeeper policy, especially in the early stage of implementation.

Some limitations should also be acknowledged in the study. First, the potential influencing factors of patients’ acceptance of policy are possibly more than those we investigated, such as patients’ experiences of referral. Second, the patients’ acceptance of the gatekeeping policy was assessed by a single item, and the acceptance-related scale with more questions should be developed in further studies. Third, our study was based on patients with GIS who may be more subjected to the policy, and therefore, the conclusion should be cautious to apply to populations with other health insurance schemes. Finally, the study did not involve the reasons why the mandatory reimbursement provision affects the patients’ acceptance. Therefore, more studies are needed to include more potential factors influencing patient acceptance with the gatekeeper policy, especially those factors for which specific interventions could be devised to determine how the mandatory reimbursement provision influence patient acceptance, and then to improve patients’ acceptance of the policy.

## Conclusion

Gatekeeper policy along with health insurance schemes is important in orienting patients towards community health facilities, and patients’ acceptance of the policy is a key factor in the implementation of the policy. Our results suggest that improvement of medical care and organization of care contributes to improve patients’ acceptance of gatekeeper policy, however, the mandatory reimbursement provision which affects patients’ acceptance should be paid more attention by policy-makers.

## Additional file

Additional file 1: The detailed contents of the EUROPEP questionnaire. (DOC 29 kb)

## Abbreviations

AGP: Patients who accept the gatekeeper policy
CHC: Community Health Centers
CHS: Community Health Service
CI: Confidence Interval
EUROPEP: European Patients Evaluate General/Family Practice
GIS: Government Insurance Scheme
NAGP: Patients who do not accept the gatekeeper policy
OR: Odds Ratio
SMI: Social Medical Insurance
